# Human Milk Oligosaccharide Composition Is Associated With Excessive Weight Gain During Exclusive Breastfeeding—An Explorative Study

**DOI:** 10.3389/fped.2019.00297

**Published:** 2019-07-18

**Authors:** Melanie W. Larsson, Mads V. Lind, Rikke Pilmann Laursen, Chloe Yonemitsu, Anni Larnkjær, Christian Mølgaard, Kim F. Michaelsen, Lars Bode

**Affiliations:** ^1^Department of Nutrition, Exercise, and Sports, University of Copenhagen, København, Denmark; ^2^Department of Nursing and Nutrition, University College Copenhagen, København, Denmark; ^3^Department of Pediatrics and Larsson-Rosenquist Foundation Mother-Milk-Infant Center of Research Excellence, University of California, San Diego, La Jolla, CA, United States

**Keywords:** growth, obesity, infancy, breastfeeding, human milk, human milk oligosaccharides, infant feeding, infant

## Abstract

**Background:** Some infants experience excessive weight gain during exclusive breastfeeding. The cause is unknown, but variation in human milk composition might play a role. Several human milk koligosaccharides (HMOs) have been associated with growth velocity in breastfed infants, and it has been suggested that the mechanism could be through an effect on infant gut microbiota composition.

**Objective:** The purpose of this exploratory study was to evaluate if HMO composition was different in milk fed to infants with excessive weight gain compared to infants with normal weight gain. Furthermore, we aimed to examine if HMO composition was associated with growth velocity and change in body composition and if there were maternal determinants of HMO composition.

**Materials and Methods:** We recruited 13 high weight-gain (HW) and 17 normal weight-gain (NW) breastfed infants, collected human milk and anthropometry data at 5 and 9 months, and analyzed HMO composition by high performance liquid chromatography.

**Results:** In the HW group eight out of 11 infants received milk from secretor mothers and in the NW group 15 out of 17. Comparing milk from Secretor mothers only, four HMO's were significantly different between the HW and NW group at 5 months and two remained significant at 9 months. Total HMO concentrations as well as total HMO-bound fucose at 5 months were positively associated with both fat mass index (FMI) and weight velocity from 0 to 5 months (all *p* < 0.025). 2′-fucosyllactose (2′-FL) was positively associated with weight velocity from 0 to 5 months and FMI at 5 months. In contrast, lacto-N-neotetraose was lower in the HW group (*p* = 0.012) and negatively associated with height-for-age Z-scores (*p* = 0.008), weight velocity from 0 to 5 months (*p* = 0.009) and FMI (*p* = 0.033). Maternal BMI at 5 months was negatively associated with 6′-sialyllactose and sialyl-lacto-N-tetraose (LSTb) and positively with 2′-FL, total HMO and total HMO-bound fucose (all *p* ≤ 0.03).

**Conclusion:** In a small cohort, we found significantly different HMO concentrations in milk to exclusively breastfed infants with excessive weight gain, suggesting that some HMOs, including 2′-FL, which is the most abundant HMO and currently added to some infant formula, could be part of the cause for the excessive weight gain.

## Introduction

Human milk is recommended as the optimal nutrition for infants due to a wide range of beneficial effects for both mother and infant (1). This includes a potential protective effect against later overweight and obesity for the child, even though not all studies support this view (1–3). The conflicting findings may be partially due to the diverse composition of human milk, which contains macronutrients, micronutrients, and a host of bioactive compounds, some of which seem to affect growth and body composition (4, 5). Human milk oligosaccharides (HMOs) have recently been linked to growth in early infancy. This was observed in 37 mother-infant dyads where an increase in lacto-N-fucopentaose (LNFP) I was associated with lower infant weight at 1 and at 6 months, and lower lean and fat mass at 6 months. Further, LNFP II and disialyl-lacto-N-tetraose (DSLNT) were associated with higher fat mass at 6 months (6). Adding HMOs to infant formula has also gained interest because of the potential positive effects on the microbiota and the immune system of the infant. An intervention study adding the HMOs 2′-fucosyllactose (2′-FL) and lacto-N-neotetraose (LNnT) to infant formula showed lower morbidity and no effect on growth (7). Little is known about which factors determine the variability in HMO concentration, however, single nucleotide polymorphisms in the fucosyltransferase 2 (FUT2) secretor gene result in human milk that is deficient in α1,2-fucosylated oligosaccharides. Women with an active FUT2 enzyme are referred to as Secretors as they secrete a substantial amount of α1,2-fucosylated oligosaccharides; women with an inactive FUT2 that lack α1,2-fucosylated oligosaccharides are referred to as Non-secretors.

Some exclusively breastfed infants have an excessive weight gain in the first 6 months of life, but it is not known if the risk of later obesity is lower in this group of infants compared to infants breastfed less. Only a few studies have targeted this group of infants and therefore little is known about the causes for this excessive weight gain (8–12). Since the high weight gain occurs during the exclusive breastfeeding period and some studies show a noticeable catch-down after introduction to complementary food (9–11), it is reasonable to search for answers for this growth pattern in human milk composition itself. We have recently shown that infants with excessive weight gain during exclusive breastfeeding in the SKOT III cohort had a marked catch-down in weight and BMI z-scores from 5 to 9 months, when complementary foods were introduced (11).

Since some HMOs are related to growth, we hypothesized that differences in HMO composition could be part of the explanation for the early excessive weight gain seen in some exclusively breastfed infants. To test this hypothesis, we analyzed HMO composition in milk samples from the exploratory SKOT III cohort of exclusively breastfed infants with excessive weight-gain and compared the values with a group of exclusively breastfed infants with normal weight-gain.

## Materials and Methods

### Study Design and Subjects

The mother-infant dyads were part of an ongoing prospective observational cohort study, the SKOT III cohort. The cohort included mothers and their 4–6 months old infants in two groups based on weight-for-age z-scores (WAZ). Infants with a WAZ > 2 and an increment of > +1 SDS in WAZ during the first 5 months post-partum, were recruited to a high weight-gain group (HW-group); infants with a WAZ between −1.0 and +1.0 SD were recruited to a normal weight-gain (NW) group. Further inclusion criteria for both groups were exclusive or full breastfeeding to at least 4 months post-partum. Infants in both groups were examined at age 5–6½ months and 9 months ± 2 weeks. Recruitment and overall study design has previously been described in detail together with human milk intake, content of macronutrients and hormones, as well as infant growth and body composition in the two groups (11). We did not match the infants based on sex, birth weight, maternal age, parity, and mode or place of delivery, however, the groups were overall well-matched.

The study protocol was approved by the Regional Ethical Committee of the Capital Region of Denmark in accordance with the Helsinki declaration (H-15008948) and the Data Protection Agency (2015-57-0117 & 2015-57-0116) and written informed consent was obtained from all parents.

Seventeen infants were referred according to the WAZ inclusion criteria to the HW group. However, four infants were excluded since they did not experience excessive weight gain although they had a WAZ > 2SDS at the first examination. Two infants were excluded because they had a high length and therefore a BAZ < 2.00 and two were excluded because they had a relatively low weight gain from birth to 5 months (their birthweights were above 4.00 kg and changes in WAZ from birth to 5 months were −0.16 and +0.6 and could therefore not be defined as excessive weight gain). Thus, the HW-group included 13 infants with at least + 1.0 SDS increment in WAZ during the first 5–6 months post-partum. In the NW-group 42 parents showed interest in participating. Of these, 19 mother-infant dyads fulfilled the inclusion criteria. However, two infants were excluded. One had a low birth weight (2.67 kg; WAZ −1.3 SDS) followed by a catch-up with a WAZ increment of 1.8 SDS. The other one had a birth weight of 4.66 kg (WAZ +2.5 SDS) followed by a catch down in WAZ of−2.9 SDS. Thus, the NW-group included 17 infants with an increment in WAZ during the first 5–6 months post-partum within normal range, defined as <0.67 SDS.

### Anthropometry

Anthropometric measurements have been described in detail previously (11). In brief, weight and length were measured using standard procedures. Body composition was measured using Bioelectrical Impedance Analyzer (BIA) Quantum III (RJL Systems, Michigan, USA) and fat free mass (FFM), fat mass (FM), and fat mass percentage (FM%) were then calculated using the Lingwood Equation (11, 13). Maternal pre-pregnancy BMI as well as gestational weight gain were self-reported while maternal weight and height were measured using standardized procedure at the infant's age 5 months visit (11).

### 24 h Milk Volume

The 24 h milk intake has been explained in detail previously (11). To measure the 24 h milk intake at 5 months, mothers weighed their infants for a period of 72 h before and after each breastfeeding-session (each feed) using an electronic baby weighing scale (Tanita BD 815 MA, Tanita Corporation, Tokyo, Japan). Calculation of intake in grams was done by subtracting weight of the infant before the feed from the weight after the feed. In cases where test weighing was not completed for all feeds, the intake was estimated using an average of intake per feed calculated from the mother's registration. No correction for infant insensible water loss was made, and therefore the milk intake is likely to be underestimated by 3–10% (14, 15).

### Human Milk Sampling and HMO Analysis

Mothers were asked to pump the entire content of both breasts using a manual breast pump (Type Harmony™, Medela AG, Baar, Switzerland) at infant age 5–6½ months and 9 months. The milk samples were stored at −20°C in the homes of the participants and transported in a bag with an ice pack and stored at −80°C at the University of Copenhagen. HMOs were analyzed in well-mixed samples of right and left breast at the University of California, San Diego, using high performance liquid chromatography (HPLC) after fluorescent derivatization (16). In brief, raffinose was added to the milk samples as an internal standard to allow for absolute HMO quantification. Oligosaccharides were isolated by solid phase extraction (SPE) over C18 and carbograph microcolumns, derivatized with 2-aminobenzamine (2AB), and further purified over silica gel SPE microcolumns. 2AB-labeled HMOs were analyzed by HPLC on an amide column with fluorescent detection. HMOs were annotated based on retention time and offline mass spectrometry and quantified based on standard response curves and in relation to the internal standard. Secretor status was determined based on presence or near-absence of 2′-FL and lacto-N-fucopentaose I. Since overall HMO composition differs between Secretors and Non-secretors, HMO were compared between the HW and NW for Secretors and Non-secretors combined as well as stratified by secretor status. However, as there was only a very few infants in the Non-secretor group this was not examined separately. HMO concentrations per mL were multiplied with total 24 h milk intake volume to yield absolute HMO intake per 24 h. To assess the overall diversity of HMO composition, Simpson's Diversity index was calculated as the reciprocal sum of the square of the relative abundance of each HMO.

### Statistical Analysis

Statistical analyses were performed using the R statistical environment (http://cran.r-project.org/, version 3.4.0). Descriptive results are presented as means and SD, mean and range or median and interquartile range (IQR: 25th and 75th percentile) as appropriate.

Differences between groups baseline characteristics was analyzed by Fisher's exact Chi-squared test for proportions, by independent *t*-test for age at visit, and by linear model adjusted for sex for all other variables. Mann-Whitney *U*-test was used to test differences between the two study groups, as HMO concentrations were not normally distributed. Associations between the different HMOs and infant WAZ, BMI-for-age z-scores (BAZ) and height-for-age z-scores (HAZ), fat mass index (FMI), fat free mass index (FFMI) and weight velocity from birth to 5 months as well as associations between mothers BMI, gestational weight gain and HMOs were investigated by Spearman's correlation, for the groups combined. We did not adjust for multiple confounders since this was not feasible given the small sample size. We used both raw and Benjamini-Hochberg false discovery rate–corrected *P*-values. In line with the explorative nature of the work we treated results with a raw *P* < 0.05 as being of interest. None of the false discovery rate–corrected *P*-values were <0.05 and we have only reported raw *p*-values.

## Results

### Mother and Child Characteristics

Infants in the HW group weighed ~450 g more and were 1.3 cm longer at birth than the NW group (Table 1). From birth to the 5 months visit weight gain was excessive in the HW group with about 100 g/week more than the NW group (*p* < 0.001). From the 5 to the 9 months visit weight gain per week was the same in the two groups (about 80 g/week, Table 1), but WAZ decreased in the HW group and was unchanged in the NW group. The BAZ decreased from 5 to 9 months from 2.49 to 2.06 in the HW group. The mothers in the two groups were of the same age and the same percentage was primipara (Table 1). The mothers in the HW group had higher body weight pre-pregnancy and at 5 months, but there was no difference in BMI either pre-pregnancy or at 5 months. The mean duration of exclusive or full breastfeeding for the HW and NW group, were 5.14 months (range: 3.69–6.46) and 5.54 months (range: 3.46–6.46), respectively (*p* = 0.29). At 9 months 83% of the infants in the HW and 94% in the NW group were still breastfed (*p* = 0.56).

**Table 1:** Maternal and child characteristics according to high weight gain (HW) or normal weight gain (NW) group^a^.

***Infant Characteristics***	**HW group**	**NW group**	***p-*value^b^**
At birth			
*N*	13	17	
Gender (girls) n(%)	5 (38.5)	10 (58.8)	0.27
Gestational age, weeks	40.7 (40.5, 41.1)	40.4 (39.7, 41.3)	0.42
Cesarean delivery, n(%)	4 (33.3)	2 (11.8)	0.20
Weight, kg	3.99 ± 0.41	3.55 ± 0.31	**0.006**
Length, cm	53.0 ±1.4	51.7 ±1.7	**0.045**
Weight-for-age z-score	1.32 ± 0.75	0.54 ± 0.61	**0.006**
BMI-for-age z-score	0.56 ± 0.80	−0.08 ± 0.59	**0.025**
5 month visit			
*N*	13	17	
Age, months	5.6 ±0.5	5.9 ±0.3	0.054
Weight, kg	10.60 ±0.88	7.89 ±0.50	** <0.001**
Length, cm	70.5 ±2.2	68.1 ±2.1	**0.009**
Weight-for-age z-score	3.02 ± 0.76	0.39 ± 0.55	** <0.001**
BMI-for-age z-score	2.49 ± 0.99	−0.09 ± 0.83	** <0.001**
Fat mass percentage, %	35.40 ± 3.18	27.78 ± 3.14	** <0.001**
Weight velocity birth to 5 month visit, g/w	271.9 ±32.4	169.2 ±17.1	** <0.001**
WAZ change from birth to 5 month visit	1.71 ± 0.69	−0.15 ± 0.68	** <0.001**
24 h milk intake, g	981.9 (653.8–1321.4)	852.0 (482.7–1234.7)	0.19
9 month visit			
*N*	12	17	
Age, months	9.0 ±0.3	9.1 ±0.2	0.48
Weight, kg	11.65 ±0.79	9.03 ±0.37	** <0.001**
Length, cm	75.7 ±1.9	72.5 ±2.0	** <0.001**
Weight-for-age z-score	2.63 ± 0.56	0.47 ± 0.49	** <0.001**
BMI-for-age z-score	2.06 ± 0.89	0.15 ± 0.80	** <0.001**
Fat mass percentage, %	36.12 ± 3.50	31.02 ± 2.26	** <0.001**
Weight velocity 5 to 9 month visit, g/w	80.4 ±22.47	81.9 ±20.76	0.93
WAZ change from 5 to 9 month visit	−0.30 ± 0.34	0.08 ± 0.29	**0.004**
*Maternal Characteristics*			
Age, years	32.5 ±3.9	33.7 ±3.2	0.40
Parity, >1 (%)	53.9	52.9	0.96
Pre-pregnancy weight, kg	71.9 ±12.0	60.6 ±6.0	**0.002**
Height, cm	170.2 ±5.5	165.8 ±7.3	0.077
Pre-pregnancy BMI, kg/m^2^	24.2 (22.1, 26.6)	22.2 (21.2, 22.5)	0.082
Gestational weight gain, kg	14 (12, 17)	15 (13, 18)	0.400
Weight, kg at 5 months	75.7 ±16.8	62.2 ±5.5	**0.004**
Weight, kg at 9 months	70.9 ±15.9	61.9 ±5.9	0.053

a *Values are expressed as mean ± standard deviation, median (25th, 75th percentile) or number (percentage) as appropriate. HW, high weight-gain group; NW, normal weight-gain group*.

b *p-values for differences between groups analyzed by Fisher's exact Chi-squared test for proportions, by independent t-test for age at visit, and by linear model adjusted for sex for all other variables. Bold values indicate significance at a P < 0.05 level*.

Mean 24 h milk intake was 130 g (15%) higher in the HW group, but the difference was not significant (Table 1) and there was no difference in human milk intake per kg per day between the groups (data not shown). At the 5 months visit 73% of the infants in the HW group and all infants in the NW group were introduced to complementary feeding (*p* = 0.064). The median contribution of complementary feeding to the total energy intake did not differ between the HW group and the NW group (15.9 vs. 23.0%, *p* = 0.37).

### Differences in HMO Composition Between HW and NW Groups

Human milk oligosaccharide (HMO) composition in Secretor and Non-secretor mothers differ significantly (17). Thus, HMO data were compared between the HW and NW; first stratified by secretor status and subsequently for Secretors and Non-secretors combined. Eight out of the 11 mothers with infants in the HW group and 15 of the 17 mothers with infants in the NW group were Secretors. As there were only five Non-secretors in total, we did not do separate analysis for these.

Differences in HMO content between the HW and NW groups at 5 and 9 months in milk from Secretor mothers alone are shown in Table 2. At 5 months, total HMO-bound fucose (*p* = 0.033) and DFLac (*P* = 0.045) were higher in the HW group compared to the NW group, whereas DFLNH (*p* = 0.045) and LNnT (*p* = 0.012) were lower in the HW group. No differences were observed for other HMOs. Total HMO-bound fucose remained significantly higher and LNnT concentrations remained significantly lower in the HW group at 9 months compared to the NW group, whereas no differences were observed for other HMOs. The most abundant HMO, 2′-FL was 36% higher in the HW group, but the difference was only borderline significant (*p* = 0.061). Overall, the results were similar between testing absolute concentrations and relative abundance of HMOs (data not shown).

**Table 2:** Human milk oligosaccharides content and 24 h intake according to high weight gain (HW) or normal weight gain (NW) groups, including only Secretors at 5 and 9 months^a^.

**HMOs**	**Concentration (nmol/ml)**			**Absolute intake (mg/d)**		
	**HW**	**NW**	***p*-value**	**HW**	**NW**	***p*-value**
**Secretor**, ***n*** **yes (%)**						
5 mo	8 (72)	15 (87)				
9 mo	6 (75)	12 (86)				
**Fucosylated or sialylated lactose**						
2′-FL						
5 mo	8360 (7045, 10276)	6126 (5112, 7000)	0.061	3885 (2933, 5460)	2360 (1987, 3693)	0.096
9 mo	8201 (6269, 10962)	5191 (4228, 7598)	0.160			
3-FL						
5 mo	479 (422, 676)	470 (355, 577)	0.519	226 (187, 331)	191 (142, 298)	0.346
9 mo	709 (673, 773)	672.86 (400.55, 920.70)	0.851			
DFLac						
5 mo	888 (850, 1005)	781 (593, 868)	**0.045**	628 (421, 725)	389 (267, 567)	0.082
9 mo	1221.27 (945.75, 1587.07)	978.96 (769.49, 1358.50)	0.223			
3′-SL						
5 mo	896 (818, 1030)	777 (614, 1042)	0.401	556 (413, 717)	407 (318, 584)	0.218
9 mo	1104 (1004, 1373)	1125 (700, 1716)	0.851			
6′-SL						
5 mo	21 (193, 246)	247 (227, 315)	0.071	145 (116, 157)	128 (105, 163)	0.942
9 mo	102.51 (87.59, 205.27)	148.70 (116.79, 207.36)	0.512			
**Non-fucosylated, non-sialylated HMOs**						
LNT						
5 mo	1109 (768, 1475)	1340 (1064, 1511)	0.333	845 (637, 915)	700 (555, 922)	0.942
9 mo	598 (477, 669)	706 (502, 1344)	0.399			
LNnT						
5 mo	592 (525, 649)	817 (689, 974)	**0.012**	399 (330, 509)	438 (380, 620)	0.311
9 mo	424 (365, 543)	736 (504, 1061)	**0.049**			
LNH						
5 mo	84 (74, 96)	101 (76, 144)	0.175	76 (62, 108)	91 (80, 122)	0.772
9 mo	43 (41, 87)	43 (33, 62)	0.512			
**Fucosylated, non-sialylated HMOs**						
LNFP I						
5 mo	680 (552, 894)	932 (449, 1782)	0.846	585 (411, 708)	956 (286, 1184)	0.612
9 mo	723 (576, 786)	684 (470, 1258)	0.925			
LNFP II						
5 mo	1656 (1496, 2251)	1926 (1415, 2217)	0.796	1553 (1180, 1790)	1191 (909, 1696)	0.515
9 mo	1436 (1291, 1535)	1445 (1220, 2154)	0.708			
LNFP III						
5 mo	68 (59, 85)	91 (77, 132)	0.071	65 (47, 80)	65 (58, 83)	0.717
9 mo	82 (65, 96)	96 (84, 115)	0.160			
DFLNT						
5 mo	1565 (1410, 1601)	1486 (1422, 1720)	0.949	1354 (1140, 1622)	1213 (994, 1489)	0.247
9 mo	1863 (1531, 1992)	1827 (1590, 2054)	0.851			
FLNH						
5 mo	36 (26, 40)	39 (32, 69)	0.197	40 (31, 46)	40 (35, 59)	0.515
9 mo	16 (16, 19)	17 (10, 27)	1.000			
DFLNH						
5 mo	15 (13, 20)	25 (17, 50)	**0.045**	20 (18, 26)	36 (17, 54)	0.070
9 mo	14 (13, 18)	15 (12, 29)	0.640			
**Non-fucosylated, sialylated HMOs**						
LSTb						
5 mo	111 (80, 159)	149 (115, 160)	0.333	111 (88, 132)	123 (80, 145)	0.612
9 mo	132 (70, 208)	158 (148, 182)	0.512			
LSTc						
5 mo	31 (12, 33)	33 (15, 42)	0.175	23 (11, 35)	26 (14, 31)	1.000
9 mo	12.67 (8.11, 17.41)	8.62 (6.29, 16.38)	0.851			
DSLNT						
5 mo	275 (213, 389)	311 (253, 375)	0.606	308 (265, 512)	293 (262, 481)	0.664
9 mo	413 (252, 553)	395 (372, 437)	0.851			
DSLNH						
5 mo	24 (16, 43)	37 (20, 52)	0.220	43 (26, 57)	59 (18, 71)	0.385
9 mo	4 (4, 7)	15 (8, 27)	0.190			
**Fucosylated, sialylated HMOs**						
FDSLNH						
5 mo	184 (135, 257)	326 (168, 404)	0.061	337 (223, 537)	503 (225, 586)	0.515
9 mo	139 (12, 158)	223 (147, 367)	0.111			
**HMO-bound Fucose**						
5 mo	16580 (15740, 17856)	14981 (14196, 15786)	**0.033**			
9 mo	18115 (16353, 19416)	14994 (14137, 17079)	**0.049**			
**HMO-bound Sialic acid**						
5 mo	2176 (1993, 2678)	2743 (2276, 2974)	0.366			
9 mo	2413 (2135, 2989)	2784 (2583, 3378)	0.261			
**HMO sum**						
5 mo	17602 (17022, 18474)	16584 (15656, 17199)	0.061	11039 (8707, 14336)	9832 (8054, 11430)	0.277
9 mo	17771 (16462, 19312)	15623 (15134, 17773)	0.061			
**Diversity**						
5 mo	3.92 (3.06, 4.67)	5.48 (4.85, 5.92)	0.061			
9 mo	4.10 (2.98, 5.19)	6.01 (4.39, 6.78)	0.160			

a *Data is presented for Secretors only as median (IQR) and tested using Mann-Whitney U-test. mo, months; 2′-FL, 2′-fucosyllactose; 3-FL, 3-fucosyllactose; 3′-SL, 3′-sialyllactose; 6′-SL, 6′-sialyllactose; LNT, lacto-N-tetraose; LNnT, lacto-N-neotetraose; LNFP I-II-III, lacto-N-fucopentaose; LSTb, LSTc, sialyl-lacto-N-tetraose; DSLNT, disialyl-lacto-N-tetraose; DFLac, difucosyl-lactose; DFLNT, difucosyl-lacto-N-tetraose; LNH, lacto-N-hexaose; FLNH, fucosyl-lacto-N-hexaose; DFLNH, difucosyl-lacto-N-hexaose; FDSLNH, fucosyl-disialyl-lacto-N-hexaose; DSLNH, disialyl-lacto-N-hexaose; HMOs, human milk oligosaccharides. Bold values indicate significance at a P < 0.05 level*.

At 5 months, when combining Secretors and Non-secretors, LNnT concentrations were lower in the HW group (*P* = 0.046), similarly as for Secretors only (Supplemental Table 1). Additionally, LSTc concentrations (*P* = 0.041) and HMO diversity (*P* = 0.041) were lower in the HW group compared to the NW group. No differences were observed at 9 months.

When also considering the 24 h milk volume, there were no differences between the groups for absolute intake of HMOs (mg/d) at 5 months.

### Associations Between HMO Composition and Growth

Associations between HMO composition and anthropometry at 5 months and weight velocities from birth to 5 months were analyzed by combining the HW and NW group and excluding Non-secretors (Tables 3–5). Additional analyses were done combining Secretors and Non-secretors (Supplemental Tables 2–4).

**Table 3:** Spearman correlations between human milk oligosaccharide content and Height-for-Age Z-scores (HAZ) and BMI-for-Age Z-scores (BAZ) at 5 months (Secretors only)^a^.

**HMOs**	**HAZ**		**BAZ**	
	**Rho**	***p*-value**	**Rho**	***p*-value**
**Fucosylated or sialylated lactose**				
2′-FL	0.238	0.275	0.388	0.067
3-FL	0.221	0.311	0.209	0.338
DFLac	0.501	**0.015**	0.128	0.562
3′-SL	0.461	**0.027**	−0.014	0.948
6SL	−0.222	0.309	−0.438	**0.036**
**Non-fucosylated, non-sialylated HMOs**				
LNT	−0.217	0.319	−0.225	0.301
LNnT	−0.541	**0.008**	−0.330	0.125
LNH	0.047	0.83	−0.221	0.311
**Fucosylated, non-sialylated HMOs**				
LNFP I	−0.296	0.171	0.051	0.818
LNFP II	0.139	0.527	−0.046	0.833
LNFP III	−0.290	0.180	−0.248	0.255
DFLNT	0.218	0.317	−0.115	0.602
FLNH	−0.097	0.660	−0.340	0.112
DFLNH	−0.475	**0.022**	−0.403	0.057
**Non-fucosylated, sialylated HMOs**				
LSTb	−0.186	0.396	−0.040	0.856
LSTc	−0.334	0.119	−0.202	0.355
DSLNT	−0.176	0.422	0.040	0.856
DSLNH	0.006	0.977	−0.329	0.126
**Fucosylated, sialylated HMOs**				
FDSLNH	−0.020	0.927	−0.343	0.109
HMO-bound fucose	0.374	0.079	0.357	0.094
HMO-bound sialic acid	0.121	0.582	−0.135	0.539
HMO sum	0.230	0.290	0.379	0.074
Diversity	−0.240	0.271	−0.378	0.075

a *Data are Spearman's correlation coefficient Rho and p-values. mo, months; 2′-FL, 2′-fucosyllactose; 3-FL, 3-fucosyllactose; 3′-SL, 3′-sialyllactose; 6′-SL, 6′-sialyllactose; LNT, lacto-N-tetraose; LNnT, lacto-N-neotetraose; LNFP I-II-III, lacto-N-fucopentaose; LSTb, LSTc, sialyl-lacto-N-tetraose; DSLNT, disialyl-lacto-N-tetraose; DFLac, difucosyl-lactose; DFLNT, difucosyl-lacto-N-tetraose; LNH, lacto-N-hexaose; FLNH, fucosyl-lacto-N-hexaose; DFLNH, difucosyl-lacto-N-hexaose; FDSLNH, fucosyl-disialyl-lacto-N-hexaose; DSLNH, disialyl-lacto-N-hexaose; HMOs, human milk oligosaccharides. Bold values indicate significance at a P < 0.05 level*.

**Table 4:** Spearman correlations between human milk oligosaccharide content and weight velocity from 0 to 5 months (grams per week) (Secretors only)^a^.

**HMOs**	**Weight velocity 0–5 mo (grams pr. week)**	
	**Rho**	***p*-value**
**Fucosylated or sialylated lactose**		
2′-FL	0.500	**0.015**
3-FL	0.330	0.124
DFLac	0.417	**0.048**
3′-SL	0.261	0.229
6′-SL	−0.302	0.161
**Non-fucosylated, non-sialylated HMOs**		
LNT	−0.375	0.078
LNnT	−0.531	**0.009**
LNH	−0.134	0.541
**Fucosylated, non-sialylated HMOs**		
LNFP I	−0.102	0.644
LNFP II	−0.016	0.943
LNFP III	−0.285	0.188
DFLNT	−0.044	0.840
FLNH	−0.268	0.217
DFLNH	−0.502	**0.015**
**Non-fucosylated, sialylated HMOs**		
LSTb	−0.197	0.368
LSTc	−0.183	0.404
DSLNT	−0.142	0.517
DSLNH	−0.181	0.409
**Fucosylated, sialylated HMOs**		
FDSLNH	−0.243	0.264
HMO-bound fucose	0.565	**0.005**
HMO-bound sialic acid	−0.042	0.847
HMO sum	0.496	**0.016**
Diversity	−0.468	**0.024**

a *Data are Spearman's correlation coefficient Rho and p-values. mo, months; 2′-FL, 2′-fucosyllactose; 3-FL, 3-fucosyllactose; 3′-SL, 3′-sialyllactose; 6′-SL, 6′-sialyllactose; LNT, lacto-N-tetraose; LNnT, lacto-N-neotetraose; LNFP I-II-III, lacto-N-fucopentaose; LSTb, LSTc, sialyl-lacto-N-tetraose; DSLNT, disialyl-lacto-N-tetraose; DFLac, difucosyl-lactose; DFLNT, difucosyl-lacto-N-tetraose; LNH, lacto-N-hexaose; FLNH, fucosyl-lacto-N-hexaose; DFLNH, difucosyl-lacto-N-hexaose; FDSLNH, fucosyl-disialyl-lacto-N-hexaose; DSLNH, disialyl-lacto-N-hexaose; HMOs, human milk oligosaccharides. Bold values indicate significance at a P < 0.05 level*.

**Table 5:** Spearman correlations between human milk oligosaccharide content and fat mass index (FMI) and fat free mass index (FFMI) at 5 months (Secretors only)^a^.

**HMOs**	**FMI 5 mo**		**FFMI 5 mo**	
	**Rho**	***p*-value**	**Rho**	***p*-value**
**Fucosylated or sialylated lactose**				
2′-FL	0.468	**0.024**	0.245	0.259
3-FL	0.278	0.200	0.008	0.970
DFLac	0.280	0.196	−0.071	0.749
3′-SL	0.117	0.596	−0.247	0.256
6′-SL	−0.373	0.080	−0.338	0.115
**Non-fucosylated, non-sialylated HMOs**				
LNT	−0.304	0.158	−0.061	0.783
LNnT	−0.447	**0.033**	−0.139	0.526
LNH	−0.241	0.268	−0.294	0.173
**Fucosylated, non-sialylated HMOs**				
LNFP I	0.002	0.993	0.261	0.228
LNFP II	−0.055	0.802	−0.158	0.472
LNFP III	−0.331	0.123	−0.212	0.333
DFLNT	−0.100	0.650	−0.132	0.547
FLNH	−0.406	0.054	−0.223	0.307
DFLNH	−0.434	**0.039**	−0.192	0.381
**Non-fucosylated, sialylated HMOs**				
LSTb	−0.116	0.599	0.040	0.858
LSTc	−0.250	0.250	−0.206	0.345
DSLNT	−0.056	0.799	0.114	0.605
DSLNH	−0.323	0.133	−0.398	0.060
**Fucosylated, sialylated HMOs**				
FDSLNH	−0.342	0.110	−0.424	**0.044**
HMO-bound fucose	0.489	**0.018**	0.155	0.479
HMO-bound sialic acid	−0.129	0.556	−0.275	0.204
HMO sum	0.466	**0.025**	0.228	0.295
Diversity	−0.444	**0.034**	−0.256	0.239

a *Data are Spearman's correlation coefficient Rho and p-values. mo, months; 2′-FL, 2′-fucosyllactose; 3-FL, 3-fucosyllactose; 3′-SL, 3′-sialyllactose; 6′-SL, 6′-sialyllactose; LNT, lacto-N-tetraose; LNnT, lacto-N-neotetraose; LNFP I-II-III, lacto-N-fucopentaose; LSTb, LSTc, sialyl-lacto-N-tetraose; DSLNT, disialyl-lacto-N-tetraose; DFLac, difucosyl-lactose; DFLNT, difucosyl-lacto-N-tetraose; LNH, lacto-N-hexaose; FLNH, fucosyl-lacto-N-hexaose; DFLNH, difucosyl-lacto-N-hexaose; FDSLNH, fucosyl-disialyl-lacto-N-hexaose; DSLNH, disialyl-lacto-N-hexaose; HMOs, human milk oligosaccharides. Bold values indicate significance at a P < 0.05 level*.

**Figure 1:**
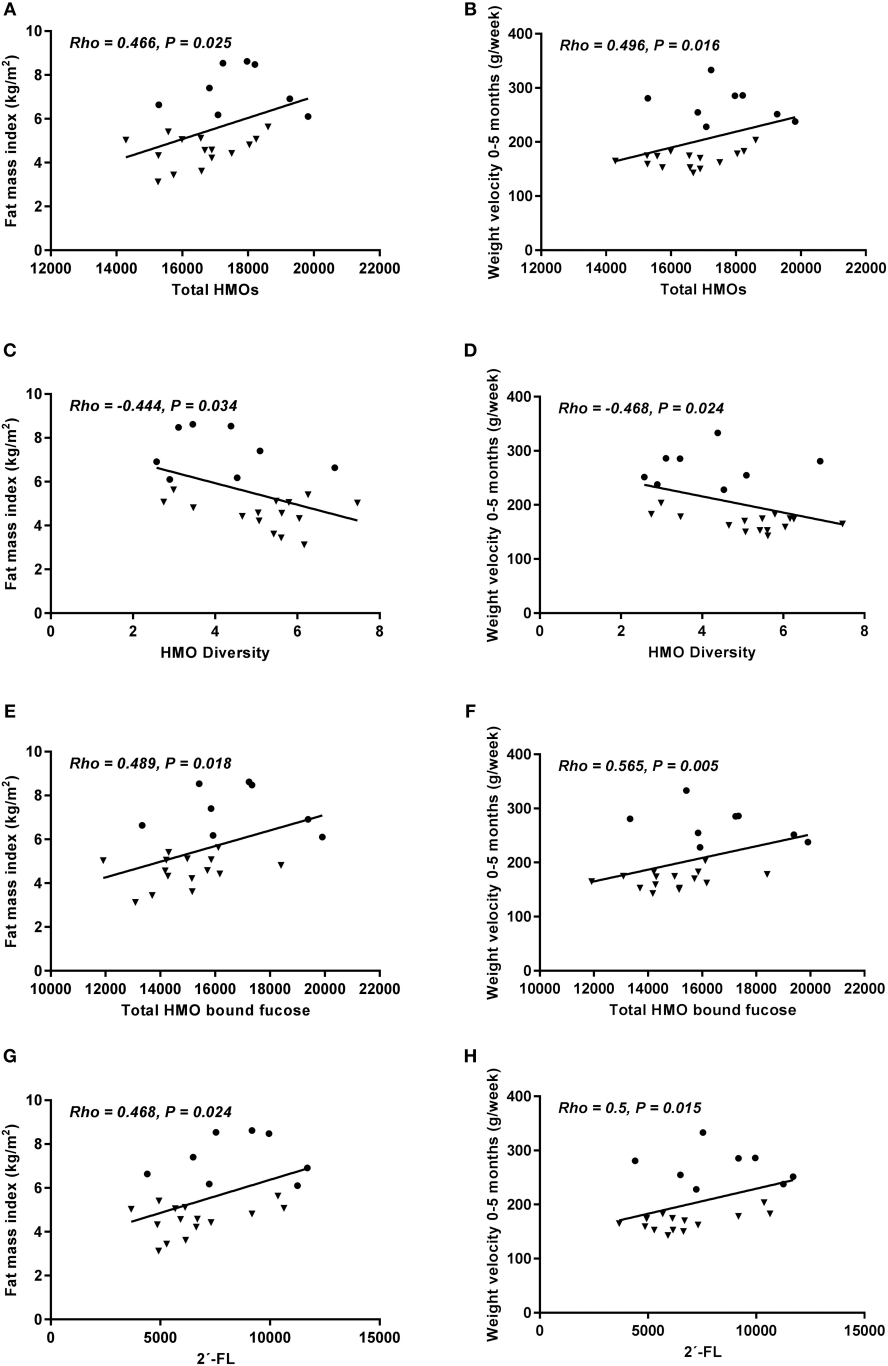
Total HMO content **(A,B)**, HMO diversity **(C,D)**, HMO-bound fucose **(E,F)**, and 2′-FL concentration **(G,H)** at 5 months in relation to fat mass index at 5 months and weight velocity 0–5 months (grams pr. week). Only secretors included. Correlations were calculated using Spearman's rank correlation, but depicted as Pearson's correlation (*n* = 23). HW = • NW = ▼.

2′-FL, the most abundant HMO, was positively associated with 0–5 months weight velocity (*p* = 0.015) and with FMI at 5 months (*p* = 0.024) (Figure 1). A similar direction for the association with change in WAZ from birth to 5 months was seen, but it did not reach statistical significance (Rho = 0.355, *p* = 0.09). DFLac was also positively associated with weight velocity (*p* = 0.048) and with length at 5 months (*p* = 0.015). 3′-SL was positively associated with length at 5 months (*p* = 0.027). 6′SL was the only HMO in the group of fucosylated or sialylated lactose HMOs which was inversely associated with anthropometry. It was inversely associated with BAZ at 5 months (*p* = 0.036) and tended to be inversely associated with FMI at 5 months (*p* = 0.08).

**Figure 2:**
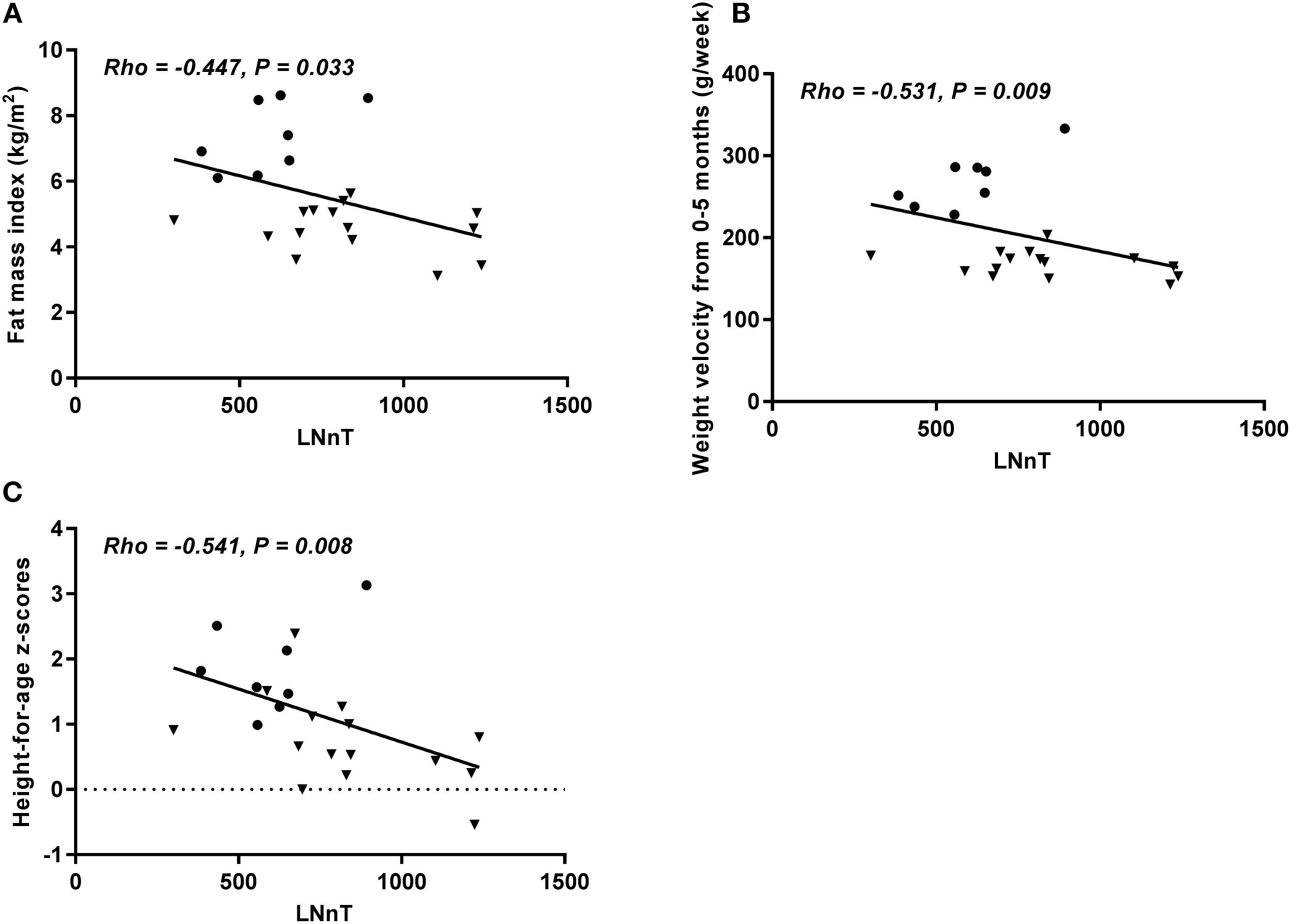
Concentration of LNnt at 5 months in relation to fat mass index at 5 months **(A)**, weight velocity from 0 to 5 months (grams pr. week) **(B)**, and height-for-age Z-score **(C)**. Only Secretors included. Correlations were calculated using Spearman's rank correlation, but depicted as Pearson's correlation (*n* = 23). HW = • NW = ▼.

In the group of non-fucosylated, non-sialylated HMOs, only LNnT was associated with anthropometry. There was an inverse association with length (*p* = 0.008), with weight velocity (*p* = 0.009) and FMI (*p* = 0.033) (Figure 2). Again a trend for an inverse association with change in WAZ from birth to 5 months was observed (Rho = −0.389, *p* = 0.07). When combining Secretors and Non-secretors, LNnT was still inversely associated with length (*p* = 0.018, Supplemental Table 2).

Total HMO-bound fucose was positively associated with weight velocity 0–5 months (*p* = 0.005) and FMI (*p* = 0.018) (Figure 1). Total HMO was positively associated with weight velocity 0–5 months (*p* = 0.016) and FMI at 5 months (*p* = 0.025), while the association between these two measures and HMO diversity were inverse (*p* = 0.024 and 0.034, respectively) (Figure 1). Both for total HMO-bound fucose and total HMO a similar direction for the association with change in WAZ from birth to 5 months was observed, but it did not reach statistical significance (Rho = 0.373, *p* = 0.08 and Rho = 0.338, *p* = 0.10, respectively).

When combining Secretors and Non-secretors, HMO diversity was still inversely associated with BAZ (*p* = 0.046), weight velocity (*p* = 0.023) and FMI at 5 months (*p* = 0.024) (Supplementary Tables 2–4).

### Maternal Determinants of HMO Content

Maternal pre-pregnancy BMI, gestational weight gain, and BMI at 5 months were examined as potential determinants of HMO concentration in human milk.

In Secretor women, none of the HMOs were significantly associated with any of these three maternal determinants (Table 6).

**Table 6:** Maternal determinants of HMO content at 5 months (Secretors only)^a^.

	**Maternal BMI (5 mo)**		**Prepregnancy BMI**		**Weightgain in pregnancy**	
**HMOs**	**Rho**	***p*-value**	**Rho**	***p*-value**	**Rho**	***p*-value**
**Fucosylated or sialylated lactose**						
2′-FL	0.336	0.117	0.279	0.198	−0.010	0.964
3-FL	0.098	0.657	0.039	0.861	0.027	0.903
DFLac	0.116	0.599	0.243	0.264	−0.326	0.129
3′-SL	−0.024	0.914	0.080	0.717	−0.149	0.498
6′-SL	−0.349	0.103	−0.271	0.211	−0.157	0.475
**Non-fucosylated, non-sialylated HMOs**						
LNT	−0.155	0.480	−0.049	0.823	0.035	0.873
LNnT	−0.123	0.578	−0.202	0.356	0.249	0.251
LNH	−0.190	0.386	−0.151	0.491	0.071	0.748
**Fucosylated, non-sialylated HMOs**						
LNFP I	0.100	0.650	−0.039	0.861	0.131	0.550
LNFP II	−0.167	0.446	−0.055	0.802	−0.132	0.549
LNFP III	−0.104	0.638	−0.139	0.526	0.216	0.322
DFLNT	−0.379	0.074	−0.225	0.301	−0.233	0.284
FLNH	−0.032	0.886	0.029	0.897	0.131	0.550
DFLNH	0.122	0.581	−0.072	0.744	0.226	0.301
**Non-fucosylated, sialylated HMOs**						
LSTb	−0.256	0.239	−0.268	0.217	0.055	0.805
LSTc	0.086	0.697	0.086	0.697	0.166	0.449
DSLNT	−0.163	0.457	−0.121	0.584	−0.001	0.996
DSLNH	−0.164	0.455	−0.003	0.989	0.062	0.777
**Fucosylated, sialylated HMOs**						
FDSLNH	−0.180	0.412	−0.155	0.480	0.072	0.744
HMO-bound fucose	0.285	0.188	0.273	0.208	−0.152	0.488
HMO-bound sialic acid	−0.299	0.165	−0.204	0.352	−0.021	0.923
HMO sum	0.374	0.079	0.308	0.152	−0.030	0.893
Diversity	−0.321	0.135	−0.266	0.220	−0.029	0.896

a *Data are Spearman's correlation coefficient Rho and p-values. mo, months; 2′-FL, 2′-fucosyllactose; 3-FL, 3-fucosyllactose; 3′-SL, 3′-sialyllactose; 6′-SL, 6′-sialyllactose; LNT, lacto-N-tetraose; LNnT, lacto-N-neotetraose; LNFP I-II-III, lacto-N-fucopentaose; LSTb, LSTc, sialyl-lacto-N-tetraose; DSLNT, disialyl-lacto-N-tetraose; DFLac, difucosyl-lactose; DFLNT, difucosyl-lacto-N-tetraose; LNH, lacto-N-hexaose; FLNH, fucosyl-lacto-N-hexaose; DFLNH, difucosyl-lacto-N-hexaose; FDSLNH, fucosyl-disialyl-lacto-N-hexaose; DSLNH, disialyl-lacto-N-hexaose; HMOs, human milk oligosaccharides*.

When combining Secretors and Non-secretors, maternal BMI at 5 months was significantly negatively associated with 6′-SL (*P* = 0.032), and LSTb (*P* = 0.03), and positively associated with 2′-FL (*P* = 0.017), total HMO (*P* = 0.015), and total HMO-bound fucose (*P* = 0.033) (Table 7). Associations with pre-pregnancy BMI were in the same directions but tended to be weaker than the associations with maternal BMI at 5 months. We did not find any associations between any HMOs and weight gain in pregnancy (A6).

**Table 7:** Maternal determinants of HMO content at age 5 months (Secretors and Non-secretors combined)^a^.

**HMOs**	**Maternal BMI (5 mo)**		**Prepregnancy BMI**		**Weightgain in pregnancy**	
	**Rho**	***p*-value**	**Rho**	***p*-value**	**Rho**	***p*-value**
**Fucosylated or sialylated lactose**						
2′-FL	0.446	**0.017**	0.389	**0.041**	0.027	0.893
3-FL	0.299	0.122	0.235	0.229	0.069	0.726
DFLac	0.301	0.120	0.346	0.071	−0.176	0.371
3′-SL	0.172	0.382	0.218	0.264	−0.025	0.901
6′-SL	−0.406	**0.032**	−0.323	0.094	−0.131	0.507
**Non-fucosylated, non-sialylated HMOs**						
LNT	−0.327	0.089	−0.230	0.238	−0.059	0.765
LNnT	−0.177	0.368	−0.224	0.251	0.117	0.552
LNH	0.078	0.692	0.078	0.692	0.155	0.431
**Fucosylated, non-sialylated HMOs**						
LNFP I	0.299	0.122	0.195	0.319	0.130	0.51
LNFP II	−0.361	0.059	−0.278	0.152	−0.161	0.413
LNFP III	−0.198	0.314	−0.211	0.28	0.097	0.624
DFLNT	−0.055	0.782	0.061	0.759	−0.161	0.414
FLNH	0.158	0.421	0.198	0.314	0.319	0.098
DFLNH	0.019	0.923	−0.131	0.507	0.203	0.300
**Non-fucosylated, sialylated HMOs**						
LSTb	−0.411	**0.030**	−0.403	**0.034**	−0.031	0.875
LSTc	0.342	0.075	0.325	0.091	0.217	0.267
DSLNT	−0.182	0.355	−0.152	0.440	−0.022	0.911
DSLNH	−0.203	0.300	−0.063	0.748	0.026	0.896
**Fucosylated, sialylated HMOs**						
FDSLNH	−0.257	0.187	−0.242	0.215	0.108	0.583
HMO-bound fucose	0.404	**0.033**	0.373	0.051	−0.054	0.787
HMO-bound sialic acid	−0.276	0.155	−0.215	0.272	0.032	0.871
HMO sum	0.455	**0.015**	0.393	**0.039**	0.019	0.925
Diversity	−0.257	0.187	−0.231	0.237	0.032	0.870

a *Data are Spearman's correlation coefficient Rho and p-values. mo, months; 2′-FL, 2′-fucosyllactose; 3-FL, 3-fucosyllactose; 3′-SL, 3′-sialyllactose; 6′-SL, 6′-sialyllactose; LNT, lacto-N-tetraose; LNnT, lacto-N-neotetraose; LNFP I-II-III, lacto-N-fucopentaose; LSTb, LSTc, sialyl-lacto-N-tetraose; DSLNT, disialyl-lacto-N-tetraose; DFLac, difucosyl-lactose; DFLNT, difucosyl-lacto-N-tetraose; LNH, lacto-N-hexaose; FLNH, fucosyl-lacto-N-hexaose; DFLNH, difucosyl-lacto-N-hexaose; FDSLNH, fucosyl-disialyl-lacto-N-hexaose; DSLNH, disialyl-lacto-N-hexaose; HMOs, human milk oligosaccharides. Bold values indicate significance at a P < 0.05 level*.

## Discussion

The aim of the study was to examine if differences in oligosaccharide composition in human milk were associated with the excessive weight gain observed in some exclusively breastfed infants, which had not been done before. We found only borderline significant differences between the HW and NW groups in total HMO concentration and HMO diversity, indicating higher total HMO and lower diversity in the HW group. However, concentrations of several individual HMOs were significantly different between the two groups suggesting that the variation in HMO composition could indeed be part of the explanation for the differences in growth between the two groups. This was further supported by the results showing that several HMOs were associated with anthropometry, growth velocity, and body composition in an analysis combining the HW and NW groups.

Our results indicate that certain HMOs might play a role in infant growth, which is in accordance with previously reported data. In a study by Alderete et al., LNFP I in mother's milk was negatively associated with infant weight at both 1 and 6 months of age, and with lean mass and fat mass (FM) at 6 months of age. In contrast, DSLNT and LNFP II were positively associated with FM at 6 months, whereas, FDSLNH and LNnT were associated with higher and lower %FM at 6 months, respectively. Further, at 6 months DSLNT was negatively associated with body length (6). The group also reported that higher HMO diversity at 1 month was associated with lower %FM and FM at 1 month. In accordance with these findings, we found HMO diversity to be inversely associated with FMI at 5 months. These findings could indicate that HMO diversity influences infant body composition during exclusive breastfeeding. Furthermore, HMO diversity was also associated with weight velocity in our study and diversity was considerably lower in the HW group (3.92 vs. 5.48), but only borderline significant. However, Sprenger et al. found no differences in infant anthropometric measurements between infants consuming human milk with low or high fucosyltransferase 2 (FUT2, secretor) genotype-associated HMO concentrations or composition in a group of 50 mother-infants dyads (18). However, their study did not analyze HMOs that are independent of FUT2.

The only HMO that was linked to growth in both our study and the Alderete study was LNnT. In both studies it seems to be related to lower FM% as Alderete et al. (6) found negatively associations with FM% and we found lower values of LNnT in the HW group, which had a significantly higher %FM compared to the NW group. Furthermore, we found a negative association of LNnT with FMI at 5 months. LNnT has been shown to alter the gut microbiota in humans, which could explain why it is associated with growth (19).

The most plausible mechanism linking HMOs and growth is that HMOs play a role in developing the infant microbiome. Without being degraded by the infant digestive system, HMOs reach distal parts of the infant's intestine where they are metabolized by the intestinal microbiota (20). The gut microbiota might potentially play a role in energy harvest from the diet and in energy storage for the host and has been associated with overweight and obesity. Studies have shown that a specific microbiome pattern can have an increased ability to harvest energy from the diet and thus accelerate growth (5, 21, 22). Other mechanisms linking HMOs and growth have also been suggested such as HMOs having direct effects on epithelial cell responses in the gut or that HMOs have systemic effects, by being absorbed (partly) intact into circulation (6).

Several studies have shown a distinctively different fecal microbiota composition in breastfed compared with formula-fed infants (23, 24). Furthermore, the composition of the fecal microbiota in breastfed infants were correlated with the HMO composition in the milk consumed in other studies (25).

There is increasing interest in adding HMOs to infant formula in order to optimize the intestinal microbiota and the development of the immune system. Since HMOs might influence energy harvest through alterations of the microbiota, HMOs might also affect growth. Therefore, there has been an interest in testing if the addition of HMOs to infant formula has an effect on growth. In a multicenter randomized trial, growth was compared between two groups of infants (age < 14 days) randomized to receive either a formula supplemented with LNnT and 2′-FL or a standard formula, from enrolment to 6 months of age (7). Weight gain was the same in the two groups and they concluded that a formula with these two HMOs added supports age-appropriate growth. Interestingly, we found that in the human milk the HW group received, LNnT was significantly lower both at 5 and 9 months, and was strongly negatively associated with weight velocity from 0 to 5 months and with length at 5 months (both *p* < 0.009) in the combined group. Furthermore, the content of the most abundant HMO, 2′-FL was 36% higher in the HW group compared to the NW group, though only borderline significant (*p* = 0.061), and positively associated with weight gain from 0 to 5 months and with FMI at 5 months. Thus, the effect on growth of 2′-FL and LNnT seems to be opposite, which might be the reason that adding both of these to a formula did not influence growth (7). However, another study adding 2′-FL to infant formula showed that infants in the intervention study had a growth pattern not different from breastfed infants and a review also concluded that adding 2′-FL or LNnT seems to support normal growth, although the data are limited (26, 27). In the two studies adding 2′-FL to formula the amount was 1.0 g/L (7, 27), which is considerable lower than the average 2′-FL concentration in our NW (~3 g/L) and HW group (~4 g/L) (Table 2). In the study adding two HMOs to infant formula (7) the amount of LNnT was 0.5 g/L, which is about the same concentrations as in human milk in our NW group.

The potential maternal determinants of HMO concentrations were examined in an analysis combining the HW and NW groups. Pre-pregnancy BMI and maternal BMI at the time of HMO sampling were associated with some HMOs whereas gestational weight gain was not. These associations were found in the analysis including both Secretors and Non-secretors whereas no associations were found in the analysis including only Secretors. We did not analyze Non-secretors separately since we only had very few infants in this group. Maternal weight and body composition have recently been linked to growth in infancy in 427 mother-infant dyads, showing that LNH and DFLNT concentrations were higher in overweight and obese mothers compared with normal weight mothers (17). In our study, where only few of the mothers were overweight or obese (11), we found that maternal BMI at 5 months was positively associated with total HMO, HMO-bound fucose as well as the most abundant HMO 2′-FL and negatively associated with LSTb and 6′-SL.

Our study has some limitations with the main limitation being the small sample size, which limits the power to detect differences between groups. Furthermore, we did not adjust for multiple testing as this is an exploratory study and our findings therefore, need to be verified in other studies. In addition, the study was a single center study further limiting the generalizability to other settings. Another limitation is that it was not possible to identify, recruit, and arrange clinical visits before the age of 5 to 6 months when growth velocity was decreasing and some infants had been introduced to complementary foods. HMO composition is likely to change during the course of lactation, as shown by Alderete et al. where 13 out of 16 analyzed HMOs changed significantly from 1 to 6 months post-partum (6) as well as in a cross-sectional study by Azad et al. (17) that revealed associations between HMO concentrations and days of lactation. Therefore, the HMO composition measured at 5–6 months might not fully reflect the HMO composition during the entire period where the excessive weight gain took place (0–5 months). If we had been able to measure HMOs when growth velocity was considerably higher, it is possible that we had found stronger associations between growth and HMOs, but this is speculative. Adjusting for other milk components such as fat, lactose, and protein content was not possible in the present study due to the small sample size, however, in an earlier study, minimal differences between the two groups were found and thus we think that this is unlikely to have affected the current results.

An additional limitation of the current study is the lack of long term follow up that would add additional information on the long-term effects of the excessive weight gain in early life as well as associations between HMOs and long-term growth patterns of children. Finally, long-term follow ups are essential for understanding the long-term consequences of excessive weight gain observed in some exclusively breastfed infants.

The strengths of the study are that we use a state of the art method for assessing HMOs in a well-characterized cohort. Although the number of participants was low, the fact that we have included infants with excessive weight gain and compared them with a group with a normal weight gain increases the chances of finding associations between HMO intake and growth. Furthermore, we have estimated data on total milk intake, which is the first study that not only looks at HMO concentrations but also computes total HMO intake considering both concentration and volume. However, it should be emphasized that milk intake was measured after the excessive weight gain occurred and is therefore subject to the same limitations as the HMO content discussed above. Future studies should be performed in larger samples with longer follow-up to identify the contribution of specific HMOs to infant growth and development. Furthermore, the link between HMOs and gut microbiota in relation to growth should be investigated simultaneously and in both human studies and experimental models to confirm a causal link. Moreover, determinants of HMO concentrations should be investigated to find potential modifiable factors, which could be targeted in interventions.

## Conclusions

In conclusion, we found significant differences between HMO concentrations in a group of exclusively breastfed infants with high weight gain compared to a group of infants with normal weight gain, which emphasizes that HMOs play an important role in infant growth. However, since this is a small explorative study it cannot proof causality, warranting further in-depth studies that investigate the role and underlying mechanisms that link variation in HMO composition to excessive weight gain. Understanding the link between HMOs and infant body composition, growth, and potential long-term consequences will be of paramount importance before individual HMOs are added to formula fed to infants.

## Data Availability

The datasets for this study will not be made publicly available because in this small cohort through the growth pattern of the individual infants it will be possible to identify the infants.

## Ethics Statement

The study protocol was approved by the Regional Ethical Committee of the Capital Region of Denmark in accordance with the Helsinki declaration (H-15008948) and the Data Protection Agency (2015-57-0117 and 2015-57-0116) and written informed consent was obtained from all parents.

## Author Contributions

MWL, AL, CM, KM, and LB: conceptualization. MWL, MVL, and RL: formal analysis. CM, KM, and LB: funding acquisition. MWL: investigation and project administration. MWL, AL, CM, KM, LB, and CY: methodology. MWL, MVL, KM, and LB: writing—original draft. MWL, MVL, RL, CY, AL, CM, KM, and LB: writing—review and editing.

### Conflict of Interest Statement

The authors declare that the research was conducted in the absence of any commercial or financial relationships that could be construed as a potential conflict of interest.
