# Corrigendum: Human Milk Oligosaccharide Composition Is Associated With Excessive Weight Gain During Exclusive Breastfeeding—An Explorative Study

**DOI:** 10.3389/fped.2019.00521

**Published:** 2020-01-22

**Authors:** Melanie W. Larsson, Mads V. Lind, Rikke Pilmann Laursen, Chloe Yonemitsu, Anni Larnkjær, Christian Mølgaard, Kim F. Michaelsen, Lars Bode

**Affiliations:** ^1^Department of Nutrition, Exercise, and Sports, University of Copenhagen, København, Denmark; ^2^Department of Nursing and Nutrition, University College Copenhagen, København, Denmark; ^3^Department of Pediatrics and Larsson-Rosenquist Foundation Mother-Milk-Infant Center of Research Excellence, University of California, San Diego, La Jolla, CA, United States

**Keywords:** growth, obesity, infancy, breastfeeding, human milk, human milk oligosaccharides, infant feeding, infant

In the original article, there was a mistake in the legend for Table 2 as published. The measurement **“nmol/L” was used instead of “nmol/ml.”** The correct legend appears below.

**Table 2:** Human milk oligosaccharides content and 24 h intake according to high weight gain (HW) or normal weight gain (NW) groups, including only Secretors at 5 and 9 months.

**HMOs**	**Concentration (nmol/ml)**	**Absolute intake (mg/d)**

The authors apologize for this error and state that this does not change the scientific conclusions of the article in any way. The original article has been updated.

