# Supplementary material for: Human Milk Oligosaccharide Composition Is Associated With Excessive Weight Gain During Exclusive Breastfeeding—An Explorative Study
Source: Front Pediatr. 2019 Jul 18;7:297. doi: 10.3389/fped.2019.00297 (PMC6657391; doi:10.3389/fped.2019.00297)
Supplement: Supplementary file 1 [file Data_Sheet_1.PDF]

Supplemental tables

Supplemental Table 1. Human milk oligosaccharides concentrations according to high weight gain (HW) or normal weight gain (NW) groups at 5 months (*n* = 28) and 9 months (*n* = 22). (Secretors and Non-secretors combined)[1.]

	HW	NW	*p*-value
**Fucosylated or sialylated lactose**			
**2´-FL**			
5 mo	7230 (2209, 9567)	5925 (4922, 6687)	0.589
9 mo	6471 (3671, 10006)	4997 (3947, 7384)	0.633
**3-FL**			
5 mo	443 (264, 568)	450 (317, 517)	0.760
9 mo	685 (345, 732)	513 (326, 905)	0.682
**DFLac**			
5 mo	864 (420, 947)	651 (532, 855)	0.466
9 mo	956 (595, 1506)	867 (635, 1270)	0.539
**3´-SL**			
5 mo	840 (567, 984)	777 (613, 1005)	0.869
9 mo	1029 (713, 1212)	897 (696, 1666)	0.785
**6´-SL**			
5 mo	226 (212, 333)	269 (228, 323)	0.384
9 mo	174 (93, 262)	160 (132, 249)	0.946
**Non-fucosylated, non-sialylated HMOs**			
**LNT**			
5 mo	1400 (884, 1953)	1339 (1068, 1528)	0.760
9 mo	648 (551, 1467)	871 (514, 1336)	0.838
**LNnT**			
5 mo	648 (557, 717)	817 (684, 857)	**0.046**
9 mo	517 (376, 712)	758 (537, 1000)	0.076
**LNH**			
5 mo	76 (61, 90)	96 (72, 141)	0.057
9 mo	44 (41, 64)	44 (36, 64)	0.539
**Fucosylated, non-sialylated HMOs**			
**LNFP I**			
5 mo	558 (338, 837)	546 (433, 1677)	0.466
9 mo	623 (334, 749)	558 (380, 1011)	0.838
**LNFP II**			
5 mo	2225 (1533, 2576)	1960 (1500 2408)	0.621

9 mo	1522 (1348, 2436)	1615 (1280, 2600)	1.000
**LNFP III**			
5 mo	83 (62, 131)	91 (73, 134)	0.335
9 mo	93 (74, 120)	102 (86, 162)	0.275
**DFLNT**			
**Table A1 continued**			
5 mo	1434 (931, 1592)	1482 (1377, 1685)	0.589
9 mo	1608 (1102, 1973)	1815 (1057, 1996)	0.682
**FLNH**			
5 mo	36 (22, 41)	38 (28, 64)	0.269
9 mo	16 (15, 20)	19 (11, 35)	0.453
**DFLNH**			
5 mo	18 (15, 26)	25 (17, 39)	0.126
9 mo	17 (14, 22)	16 (13, 33)	0.785
**Non-fucosylated, sialylated HMOs**			
**LSTb**			
5 mo	141 (90, 230)	149 (121, 164)	0.869
9 mo	195 (86, 277)	165 (150, 230)	1.000
**LSTc**			
5 mo	13 (6, 32)	31 (15, 42)	**0.041**
9 mo	13 (6, 19)	9 (5, 16)	0.682
**DSLNT**			
5 mo	292 (242, 455)	320 (256, 399)	0.981
9 mo	537 (298, 639)	398 (375, 475)	0.453
**DSLNH**			
5 mo	39 (18, 43)	46 (28, 54)	0.249
9 mo	6 (4, 26)	20 (10, 35)	0.275
**Fucosylated, sialylated HMOs**			
**FDSLNH**			
5 mo	235 (145, 349)	354 (170, 520)	0.105
9 mo	155 (129, 236)	227 (163, 388)	0.152

**HMO-bound fucose**			
5 mo	15850 (9386, 17290)	14303 (13704, 15719)	0.438
9 mo	16605 (12274, 19220)	14614 (13136, 16769)	0.375
**HMO-bound sialic acid**			
5 mo	2463 (2072, 2918)	2812 (2332, 3174)	0.384
9 mo	2853 (2208, 3271)	2897 (2610, 3385)	0.539
**HMO sum**			
5 mo	17089 (12375, 18089)	16564 (15272, 16899)	0.410

9 mo	16606 (14029, 18870)	15514 (14959, 17456)	0.339
**Diversity**			
5 mo	4.54 (3.28, 5.12)	5.61 (5.04, 5.93)	**0.041**
9 mo	5.06 (3.27, 5.51)	5.79 (4.47, 6.65)	0.172

[1]Data is presented for Secretors and Non-secretors combined as median (IQR) and tested using Mann-Whitney U test. Abbreviations: Months (mo), 2´-fucosyllactose (2´-FL), 3-fucosyllactose (3-FL), 3´-sialyllactose (3´-SL), 6´-sialyllactose (6´-SL), lacto-N-tetraose (LNT), lacto-N-neotetraose (LNnT), lacto-N-fucopentaose (LNFP I-II-III), sialyl-lacto-N-tetraose (LSTb, LSTc), disialyl-lacto-N-tetraose (DSLNT), difucosyl-lactose (DFLac), difucosyl-lacto-N-tetraose (DFLNT), lacto-N-hexaose (LNH), fucosyl-lacto-N-hexaose (FLNH), difucosyl-lacto-N-hexaose (DFLNH), fucosyl-disialyl-lacto-N-hexaose (FDSLNH), disialyl-lacto-N-hexaose (DSLNH), human milk oligosaccharides (HMOs).

Supplemental table 2. Spearman correlations between human milk oligosaccharide content and height-for-age (HAZ) and BMI-for-age (BAZ) at 5 months (Secretors and Non-secretors combined)[1].

	HAZ		BAZ	
**HMOs**	**Rho**	***p*-value**	**Rho**	***p*-value**
**Fucosylated or sialylated lactose**				
**2´-FL**	-0.082	0.677	0.184	0.35
**3-FL**	-0.093	0.639	0.044	0.826
**DFLac**	0.077	0.696	0.023	0.908
**3´-SL**	0.222	0.257	-0.060	0.761
**6´-SL**	0.073	0.712	-0.263	0.176
**Non-fucosylated, non-sialylated HMOs**				
**LNT**	0.042	0.83	0.016	0.934
**LNnT**	-0.445	**0.018**	-0.194	0.322
**LNH**	-0.093	0.639	-0.355	0.064
**Fucosylated, non-sialylated HMOs**				
**LNFP I**	-0.407	**0.032**	0.003	0.988
**LNFP II**	0.300	0.121	0.005	0.98
**LNFP III**	-0.187	0.341	-0.052	0.793
**DFLNT**	-0.013	0.947	-0.155	0.431
**FLNH**	-0.093	0.639	-0.247	0.206
**DFLNH**	-0.282	0.146	-0.348	0.069
**Non-fucosylated, sialylated HMOs**				
**LSTb**	0.117	0.555	0.062	0.756
**LSTc**	-0.406	**0.032**	-0.284	0.143
**DSLNT**	0.042	0.834	0.063	0.750
**DSLNH**	0.071	0.720	-0.308	0.111
**Fucosylated, sialylated HMOs**				
**FDSLNH**	0.057	0.771	-0.305	0.115
**HMO-bound fucose**	0.005	0.979	0.161	0.412
**HMO-bound sialic acid**	0.192	0.329	-0.172	0.380
**HMO sum**	-0.058	0.769	0.218	0.265
**Diversity**	-0.186	0.344	-0.380	**0.046**

[1]Data are Spearman's correlation coefficient Rho and p-values. Abbreviations: months (mo), 2´-fucosyllactose (2´-FL), 3-fucosyllactose (3-FL), 3´-sialyllactose (3´-SL), 6´-sialyllactose (6´-SL), lacto-N-tetraose (LNT), lacto-N-neotetraose (LNnT), lacto-N-fucopentaose (LNFP I-II-III), sialyl-lacto-N-tetraose (LSTb, LSTc), disialyl-lacto-N-tetraose (DSLNT), difucosyl-lactose (DFLac), difucosyl-lacto-N-tetraose (DFLNT), lacto-N-hexaose (LNH), fucosyl-lacto-N-hexaose (FLNH), difucosyl-lacto-N-hexaose (DFLNH), fucosyl-disialyl-lacto-N-hexaose (FDSLNH), disialyl-lacto-N-hexaose (DSLNH), human milk oligosaccharides (HMOs).

Supplemental table 3. Spearman correlations between content and weight velocity from 0-5 months (grams pr. week) (Secretors and Non-secretors combined)[1].

HMOs	Weight velocity 0-5 mo (grams pr. week)	
	Rho	*p*-value
**Fucosylated or sialylated lactose**		
**2´-FL**	0.200	0.308
**3-FL**	0.088	0.656
**DFLac**	0.077	0.698
**3´-SL**	0.001	0.996
**6´-SL**	-0.093	0.638
**Non-fucosylated, non-sialylated HMOs**		
**LNT**	-0.032	0.873
**LNnT**	-0.304	0.115
**LNH**	-0.439	**0.019**
**Fucosylated, non-sialylated HMOs**		
**LNFP I**	-0.046	0.816
**LNFP II**	-0.062	0.752
**LNFP III**	-0.059	0.765
**DFLNT**	-0.209	0.286
**FLNH**	-0.246	0.207
**DFLNH**	-0.269	0.167
**Non-fucosylated, sialylated HMOs**		
**LSTb**	0.019	0.923
**LSTc**	-0.274	0.158
**DSLNT**	-0.035	0.860
**DSLNH**	-0.299	0.122
**Fucosylated, sialylated HMOs**		
**FDSLNH**	-0.380	**0.046**
**HMO-bound fucose**	0.195	0.320
**HMO-bound sialic acid**	-0.201	0.305
**HMO sum**	0.228	0.243
**Diversity**	-0.429	**0.023**

[1] Data are Spearman's correlation coefficient Rho and p-values. Abbreviations: months (mo), 2´-fucosyllactose (2´-FL), 3-fucosyllactose (3-FL), 3´-sialyllactose (3´-SL), 6´-sialyllactose (6´-SL), lacto-N-tetraose (LNT), lacto-N-neotetraose (LNnT), lacto-N-fucopentaose (LNFP I-II-III), sialyl-lacto-N-tetraose (LSTb, LSTc), disialyl-lacto-N-tetraose (DSLNT), difucosyl-lactose (DFLac), difucosyl-lacto-N-tetraose (DFLNT), lacto-N-hexaose (LNH), fucosyl-lacto-N-hexaose (FLNH), difucosyl-lacto-N-hexaose (DFLNH), fucosyl-disialyl-lacto-N-hexaose (FDSLNH), disialyl-lacto-N-hexaose (DSLNH), human milk oligosaccharides (HMOs).

Supplemental table 4. Spearman correlations between human milk oligosaccharide content and fat mass index (FMI) and fat free mass index (FFMI) at 5 months (Secretors and Non-secretors combined)[1].

	FMI 5 mo		FFMI 5 mo	
**HMOs**	**Rho**	***p*-value**	**Rho**	***p*-value**
**Fucosylated or sialylated lactose**				
**2´-FL**	0.205	0.295	0.14	0.477
**3-FL**	0.062	0.755	-0.019	0.925
**DFLac**	0.092	0.642	-0.063	0.751
**3´-SL**	0.024	0.905	-0.205	0.295
**6´-SL**	-0.194	0.323	-0.241	0.217
**Non-fucosylated, non-sialylated HMOs**				
**LNT**	-0.033	0.868	0.080	0.686
**LNnT**	-0.293	0.130	-0.064	0.747
**LNH**	-0.356	0.063	-0.373	0.050
**Fucosylated, non-sialylated HMOs**				
**LNFP I**	-0.066	0.738	0.156	0.429
**LNFP II**	0.039	0.844	-0.098	0.621
**LNFP III**	-0.123	0.532	-0.022	0.913
**DFLNT**	-0.164	0.404	-0.144	0.466
**FLNH**	-0.284	0.143	-0.160	0.417
**DFLNH**	-0.369	0.053	-0.207	0.290
**Non-fucosylated, sialylated HMOs**				
**LSTb**	0.048	0.810	0.082	0.68
**LSTc**	-0.342	0.075	-0.233	0.232
**DSLNT**	0.015	0.941	0.110	0.576
**DSLNH**	-0.297	0.125	-0.372	0.051
**Fucosylated, sialylated HMOs**				
**FDSLNH**	-0.271	0.163	-0.372	0.051
**HMO-bound fucose**	0.220	0.261	0.074	0.710
**HMO-bound sialic acid**	-0.141	0.474	-0.248	0.204
**HMO sum**	0.246	0.206	0.162	0.409
**Diversity**	-0.425	**0.024**	-0.270	0.164

[1]Data are Spearman's correlation coefficient Rho and p-values. Abbreviations: months (mo), 2´-fucosyllactose (2´-FL), 3-fucosyllactose (3-FL), 3´-sialyllactose (3´-SL), 6´-sialyllactose (6´-SL), lacto-N-tetraose (LNT), lacto-N-neotetraose (LNnT), lacto-N-fucopentaose (LNFP I-II-III), sialyl-lacto-N-tetraose (LSTb, LSTc), disialyl-lacto-N-tetraose (DSLNT), difucosyl-lactose (DFLac), difucosyl-lacto-N-tetraose (DFLNT), lacto-N-hexaose (LNH), fucosyl-lacto-N-hexaose (FLNH), difucosyl-lacto-N-hexaose (DFLNH), fucosyl-disialyl-lacto-N-hexaose (FDSLNH), disialyl-lacto-N-hexaose (DSLNH), human milk oligosaccharides (HMOs).
